# A mutant fibrinogen that is unable to form fibrin can improve renal phenotype in mice with sickle cell anemia

**DOI:** 10.1002/jha2.204

**Published:** 2021-06-15

**Authors:** Marilou G. Narciso, Blair Hoeting, Jeanne M. James, Katherine VandenHeuvel, Md. Nasimuzzaman

**Affiliations:** ^1^ Division of Experimental Hematology and Cancer Biology Cincinnati Children's Hospital Medical Center Cincinnati Ohio USA; ^2^ Division Pediatric Cardiology Medical College of Wisconsin Milwaukee Wisconsin USA; ^3^ Pathology Core Cincinnati Children's Hospital Medical Center Cincinnati Ohio USA; ^4^ University of Cincinnati College of Medicine Cincinnati Ohio USA

**Keywords:** albuminuria, fibrinogen, kidney, nephropathy, pathology, sickle cell anemia, thrombin

## Abstract

Sickle cell anemia (SCA) causes nephropathy which may progress to kidney failure. To determine if soluble fibrinogen (Fib^AEK^) can prevent kidney damage in mice with SCA, we performed bone marrow transplantation (BMT) of Berkeley sickle mice into wild‐type fibrinogen (Fib^WT^), and Fib^AEK^ mice that bear a germ‐line mutation in fibrinogen Aα chain at thrombin cleavage site which prevents fibrin formation. We found improved albuminuria in SS Fib^AEK^ mice compared with SS Fib^WT^ mice at 12 months post‐BMT due to the reduced kidney fibrosis, ischemic lesions, and increased survival of podocytes in the glomeruli, but did not improve urine concentrating defect. Therefore, our study clarifies the distinct role of fibrinogen and fibrin in the renal pathology of SCA.

## 1 INTRODUCTION

Sickle cell anemia (SCA) causes acute chest syndrome, vascular‐occlusion‐associated severe pain episodes, chronic hemolytic anemia, inflammation, and multiple‐organ damage that cause nephropathy, pulmonary hypertension, and cardiomyopathy which reduce the lifespan of patients with SCA [1, 2]. With improved and comprehensive medical care, SCA patients now live longer and organ pathology has emerged as the major cause of death in adult patients with SCA. Sickle nephropathy, including tubular pathology, manifests as urine concentrating defect and glomerulopathy, manifests as proteinuria, specifically albuminuria that may progress to renal failure [3, 4]. However, a significant knowledge gap remains in understanding the SCA‐associated renal pathophysiology.

Fibrin(ogen) serves an important role in hemostasis and thrombosis [5]. Thrombin cleaves soluble fibrinogen and converts into insoluble fibrin which occludes blood vessels through the formation of fibrin mesh cross‐linked by factor XIII and thereby, stops excessive bleeding [5]. Fibrin(ogen) is also responsible for inflammatory processes, tissue injury, and wound healing [5]. Leukocytes interact with fibrinogen through integrin α_M_β_2_ receptors that stimulate phagocytosis, degranulation, and inflammation [6]. In our previous study, we found that the elimination of fibrinogen binding to the α_M_β_2_ receptor improves renal pathology in SCA mice [7]. However, the exact roles of soluble fibrinogen versus insoluble fibrin polymers in the disease processes were impossible to investigate due to the lack of a mouse model. To resolve this issue, Prasad *et al*. have generated the Fib^AEK^ mouse line that has a germ‐line mutation in the fibrinogen Aα chain where the thrombin cleavage site is located [8]. Therefore, thrombin cannot cleave and release fibrinopeptide‐A from this mutant fibrinogen that fails to form fibrin polymer. The Fib^AEK^ mouse carries soluble circulating fibrinogen, which shows a remarkable inability to clear *Staphylococcus aureus* intraperitoneal infection but has a significant infection dose‐dependent survival after acute peritonitis [8]. However, the role of soluble fibrinogen versus insoluble fibrin polymer in kidney damage in SCA is not investigated. Here, we have shown that Fib^AEK^ mutation improves renal phenotype in mice with SCA.

## 2 METHODS

All experiments were performed at Cincinnati Children's Research Foundation's veterinary facility with approval from the Institutional Animal Care and Use Committee. To determine the role of fibrin(ogen) in the renal pathology of SCA, bone marrow hematopoietic stem cells from Berkeley sickle (SS) mice expressing sickle hemoglobin or C57BL/6‐Ly5.1 (BoyJ) mice expressing normal hemoglobin were transplanted into irradiated 8‐ to 10‐week‐old recipient mice, wild‐type fibrinogen (Fib^WT^) or Fib^AEK^ to generate the chimeric mice: SS Fib^WT^, SS Fib^AEK^, BoyJ Fib^WT^, or BoyJ Fib^AEK^ (Figure S1A). The bone marrow transplantation (BMT) experiments were repeated three times and the chimeric mice were followed for 1 year. All methods have been described in the supplemental methods and our published article [7, 9].

## 3 RESULTS

We analyzed the complete blood count of our experimental mice at 12 months post‐BMT. As expected, we observed significantly higher reticulocyte counts in SS Fib^WT^ and SS Fib^AEK^ mice compared to the nonsickle, BoyJ Fib^WT^, and BoyJ Fib^AEK^ mice (Figure S1B) demonstrating that sickle phenotypes were successfully transmitted from the donor SCA mice to the recipient mice. The human sickle RBC's hemoglobin (HbS) chimerism in the SS Fib^WT^ and SS Fib^AEK^ mice were stable for the duration of the study (Figure S2). We found significantly lower RBC parameters in SS Fib^WT^ mice than the BoyJ Fib^WT^ mice. We did not observe any difference in RBC parameters between SS Fib^WT^ and SS Fib^AEK^ mice, both showing classic sickle RBC parameters [7]. We found significantly higher monocyte counts in SS Fib^WT^ and SS Fib^AEK^ mice compared to the BoyJ Fib^WT^ mice. WBC, neutrophils, and lymphocytes showed a trend toward higher counts in SS Fib^WT^ mice compared with the BoyJ Fib^WT^ mice, whereas platelet counts were decreased in SS mice. WBC, monocyte, and lymphocyte counts were similar except for higher neutrophil counts in SS Fib^AEK^ mice than the SS Fib^WT^ mice (Table S1).

We found a similar concentration of fibrinogen in BoyJ Fib^AEK^ mice and BoyJ Fib^WT^ mice, and SS Fib^AEK^ mice and SS Fib^WT^ mice. Also, we did not found any difference in plasma fibrinogen between SS and non‐SS mice (Figure S3).

**FIGURE 1:**
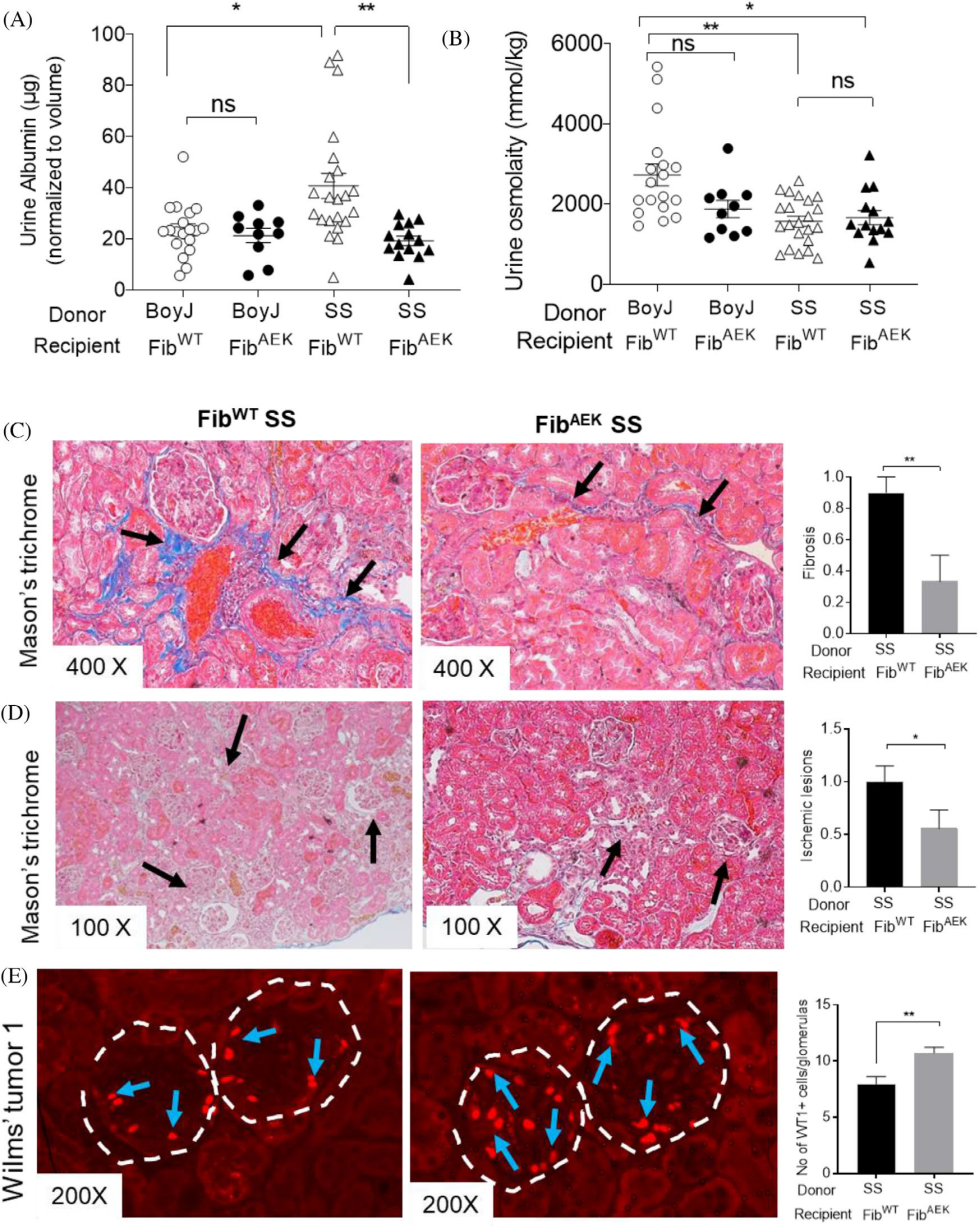
Renal function and pathology are improved in SS Fib^AEK^ mice compared with the SS Fib^WT^ mice. (A) Mouse urine albumin concentrations were measured that were normalized with 24 h urine volume. Albuminuria was increased in SS Fib^WT^ (*n* = 22) mice compared with the BoyJ Fib^WT^ (*n* = 19) mice and reduced in SS Fib^AEK^ (*n* = 14) mice compared to SS Fib^WT^ (*n* = 22) at 12 months post‐BMT. (B) SS Fib^WT^ (*n* = 22) and SS Fib^AEK^ (*n* = 14) mice had significantly diminished urine concentrating ability compared with BoyJ Fib^WT^ (*n* = 19) mice and similar urine concentrating ability between SS Fib^WT^ and SS Fib^AEK^ mice at 12 months post‐BMT. Each symbol represents an individual mouse. (C, D) Exemplary kidney sections of SS Fib^WT^ mice (*n* = 10, left panel), SS Fib^AEK^ mice (*n* = 9, middle panel), and histologic features (right panel) showing fibrosis (C) and ischemic lesions (D). Each kidney section was entirely examined and scored. Histopathology scores are ranged from 0 to 5, where 0 is the normal kidney morphology; 1 is the pathology in less than 20% of the kidney sections; 2 is the pathology in 21–40% of the kidney sections; 3 is the pathology in 41–60% the kidney sections; 4 is the pathology in 61–80% the kidney sections. (E) Immunofluorescence staining of the kidney sections shows decreased podocyte marker, Wilms’ tumor 1 expression (WT1, red dots marked with blue arrows inside white dotted circles) in SS Fib^WT^ mice (*n* = 6) compared to SS Fib^AEK^ mice (*n* = 5). Twenty glomeruli of each kidney section were counted and the average number of WT1^+^ podocytes is shown in the graph. The bars in the graphs indicate the mean ± standard error of the mean (SEM). One‐way ANOVA followed by Tukey's or Dunn's multiple comparison test for multiple groups, and Student's *T*‐test or Mann–U Whitney test for two groups were used. Statistical significance is indicated as **p, ≤0.01, *p, ≤0.05; and ns, not significant

Similar to human SCA patients, mice with SCA progressively develop nephropathy with aging [3, 7]. To assess renal function, we harvested 24‐h urine samples from our mice. We found significantly increased albuminuria in SS Fib^WT^ mice compared with BoyJ Fib^WT^ mice at 12 months post‐BMT (Figure 1A). We found significantly reduced urine albumin in SS Fib^AEK^ mice than the SS Fib^WT^ mice at 12 months post‐BMT (Figure 1A). To determine whether kidney pathology of increased severity is correlated with renal dysfunction, we analyzed the kidney histopathology of our experimental mice after euthanization [7]. Compared to the SS Fib^WT^ mice, SS Fib^AEK^ mice had significantly reduced renal fibrosis (Figure 1A), ischemic lesions (Figure 1B), and increased survival of Wilms’ tumor 1 expressing podocytes in the glomeruli (Figure 1C). We also found a trend for decreased inflammatory infiltrates in SS Fib^AEK^ mice compared to the SS Fib^WT^ mice (Figure S4). Both SS Fib^WT^ and SS Fib^AEK^ mice had diminished urine concentrating ability compared to BoyJ Fib^WT^ mice at 12 months post‐BMT (Figure 1B). However, urine concentrating ability was not improved in SS Fib^AEK^ mice compared to the SS Fib^WT^ mice at 12 months post‐BMT (Figure 1B). We did not found any difference in tubular pathology between SS Fib^WT^ and SS Fib^AEK^ mice (Figure S5), which is consistent with the urine concentrating ability data. Therefore, fibrin‐mediated clot formation is not playing a role in the urine concentrating ability in SCA. We did not found any pathology in the kidneys of the non‐SS mice (Figure S6). Collectively, our data suggest that Fib^AEK^ mutation offers protection against albuminuria development in mice with SCA but cannot improve urine concentrating defect.

## 4 DISCUSSION

Thrombin promotes macrophage recruitment via fibrin(ogen) that causes local inflammatory cytokine production [5]. Since inflammation is a prominent feature in SCA, we investigated whether the mechanism by which fibrin(ogen) causes end‐organ damage is via its macrophage binding domain, α_M_β_2_. In our previous study, we found that genetic elimination of the α_M_β_2_ binding motif of fibrinogen ameliorated kidney pathology in mice with SCA [7]. Our present study implies that thrombin activity can promote kidney damage in SCA via the conversion of fibrinogen to fibrin as an independent mechanism to fibrin(ogen)–leukocyte interactions mediated by α_M_β_2_.

Renal fibrosis is present in both SCA patients and mice as a consequence of the excessive accumulation of extracellular matrix and represents a failed wound‐healing process of the renal tissues [3]. Fibrin can induce renal fibrosis through activation of fibroblasts that proliferate and differentiate into myofibroblasts in response to increased profibrotic mediator secretion and deposition of collagen that ultimately results in fibrogenesis causing renal fibrosis that leads to chronic kidney diseases [10, 11, 12]. Fibrinogen‐deficient mice have significantly reduced interstitial fibroblasts proliferation without any difference in inflammatory infiltrates in the kidneys [11].

Our study demonstrates that Fib^AEK^ mutation improves renal fibrosis, ischemia, and protects podocytes from injury/loss resulting in reduced albuminuria in mice with SCA. Further studies will investigate the mechanisms of fibrinogen versus fibrin‐associated renal pathology in SCA to better delineate the relevant biological processes, which in turn will direct the development of novel treatment strategies for kidney diseases.

## AUTHORS CONTRIBUTION

Md Nasimuzzaman: Conceptualization (Lead), Formal analysis (Lead), Funding acquisition (Lead), Investigation (Lead), Writing‐original draft (Lead); Marilou Narciso: formal analysis (Supporting), Methodology (Supporting); Blair Hoeting: Formal analysis (Supporting), Methodology (Supporting); Jeanne James: Formal analysis (Supporting), Writing‐original draft (Supporting); Katherine VandenHeuvel: Formal analysis (Supporting).

## CONFLICT‐OF‐INTEREST DISCLOSURE

The authors do not have any conflict of interest to declare.

## Supporting information

Supporting Information

## References

[1] Darbari DS , Kple‐Faget P , Kwagyan J , Rana S , Gordeuk VR , Castro O . Circumstances of death in adult sickle cell disease patients. Am J Hematol. 2006;81(11):858‐863. 16924640 10.1002/ajh.20685

[2] Nasimuzzaman M , Malik P . Role of the coagulation system in the pathogenesis of sickle cell disease. Blood Adv. 2019;3(20):3170‐3180. 31648337 10.1182/bloodadvances.2019000193 PMC6849940

[3] Pham PT , Pham PC , Wilkinson AH , Lew SQ . Renal abnormalities in sickle cell disease. Kidney Int. 2000;57(1):1‐8. 10.1046/j.1523-1755.2000.00806.x 10620181

[4] Guasch A , Navarrete J , Nass K , Zayas CF . Glomerular involvement in adults with sickle cell hemoglobinopathies: prevalence and clinical correlates of progressive renal failure. J Am Soc Nephrol. 2006;17(8):2228‐2235. 16837635 10.1681/ASN.2002010084

[5] Szaba FM , Smiley ST . Roles for thrombin and fibrin(ogen) in cytokine/chemokine production and macrophage adhesion in vivo. Blood. 2002;99(3):1053‐1059. 11807012 10.1182/blood.v99.3.1053 PMC3150214

[6] Fan ST , Edgington TS . Integrin regulation of leukocyte inflammatory functions. CD11b/CD18 enhancement of the tumor necrosis factor‐alpha responses of monocytes. J Immunol. 1993;150(7):2972‐2980. 8095957

[7] Nasimuzzaman M , Arumugam PI , Mullins ES , James JM , Heuvel KV , Narciso MG , et al. Elimination of the fibrinogen integrin alphaMbeta2‐binding motif improves renal pathology in mice with sickle cell anemia. Blood Adv. 2019;3(9):1519‐1532. 31076408 10.1182/bloodadvances.2019032342 PMC6517666

[8] Prasad JM , Gorkun OV , Raghu H , Thornton S , Mullins ES , Palumbo JS , et al. Mice expressing a mutant form of fibrinogen that cannot support fibrin formation exhibit compromised antimicrobial host defense. Blood. 2015;126(17):2047‐2058. 26228483 10.1182/blood-2015-04-639849 PMC4616238

[9] Arumugam PI , Mullins ES , Shanmukhappa SK , Monia BP , Loberg A , Shaw MA , et al. Genetic diminution of circulating prothrombin ameliorates multiorgan pathologies in sickle cell disease mice. Blood. 2015;126(15):1844‐1855. 26286849 10.1182/blood-2015-01-625707 PMC4600020

[10] Meng XM , Nikolic‐Paterson DJ , Lan HY . TGF‐beta: the master regulator of fibrosis. Nat Rev Nephrol. 2016;12(6):325‐338. 27108839 10.1038/nrneph.2016.48

[11] Sorensen I , Susnik N , Inhester T , Degen JL , Melk A , Haller H , et al. Fibrinogen, acting as a mitogen for tubulointerstitial fibroblasts, promotes renal fibrosis. Kidney Int. 2011;80(10):1035‐1044. 21734641 10.1038/ki.2011.214

[12] Drew AF , Tucker HL , Liu H , Witte DP , Degen JL , Tipping PG . Crescentic glomerulonephritis is diminished in fibrinogen‐deficient mice. Am J Physiol Renal Physiol. 2001;281(6):F1157‐F1163. 11704568 10.1152/ajprenal.2001.281.6.F1157

