# Supplementary material for: A mutant fibrinogen that is unable to form fibrin can improve renal phenotype in mice with sickle cell anemia
Source: EJHaem. 2021 Jun 15;2(3):462–5. doi: 10.1002/jha2.204 (PMC9176143; doi:10.1002/jha2.204)
Supplement: Supplementary file 1 — Supporting Information [file JHA2-2-462-s001.pdf]

SUPPLEMENTAL MATERIALS AND METHODS:

Experimental mice

To generate a mouse carrying a mutant form of fibrinogen, the Aα chain thrombin cleavage residues $E^{P6}GGGVR^{P1}$ were mutated to $A^{P6}DDDDK^{P1}$ in C57BL/6-inbred and named $Fibrinogen^{AEK}$ (Fib^{AEK}) mice.[1] Berkeley sickle mice [Tg(Hu-miniLCRγ1GγAγδβbS) Hba0//Hba0 Hbb0//Hbb0] were used as the donor for bone marrow transplantation (BMT) of hematopoietic stem cell (HSC) that solely express human sickle hemoglobin (HbS).[2] Bone marrow HSC from Berkeley sickle (SS) mice or C57BL/6-Ly5.1 (BoyJ) mice expressing mouse normal hemoglobin were transplanted into irradiated 8 to 10-week old recipient mice, wild-type fibrinogen (Fib^{WT}) or Fib^{AEK} to generate chimeras: SS Fib^{WT}, SS Fib^{AEK}, BoyJ Fib^{WT}, or BoyJ Fib^{AEK}. The bone marrow transplantation experiments were repeated three times and the chimeric mice were followed for one year. All experiments were performed at Cincinnati Children's Research Foundation's veterinary facility with approval from the Institutional Animal Care and Use Committee.

Analyses of hematologic parameters

Complete blood count (CBC) including RBC indices, WBC, and platelet counts were accomplished using a Hemavet 950FS Auto Blood Analyzer (Drew Scientific, Waterbury, CT). Reticulocyte counts were performed by flow cytometry using the Thiazole Orange reagent (BD Biosciences, San Diego, CA).

Measurement of urine albumin and osmolality

24-hours urine samples were collected from the experimental mice using metabolic cages. A mouse albumin ELISA kit (Bethyl Laboratories, Montgomery, TX) was used to determine the concentration of urine albumin which was normalized to 24 h urine volume. Urine osmolality of the same samples was measured using Vapro Pressure Osmometer 5600 (Wescor Biomedical Systems, Logan, UT).[3]

Histopathology analyses of organ samples

All of the mice were weighed before being euthanized. Kidneys were fixed in 10% buffered formalin and embedded in paraffin. Sections (4 µm) were stained with hematoxylin and eosin (H&E), periodic acid-Schiff (PAS), and Masson's trichrome and evaluated by a pathologist blinded to the animal genotypes.[3]

Immunofluorescent staining of kidney sections for podocyte marker, Wilms tumor (WT) 1

Formalin-fixed, paraffin-embedded kidney sections (4 µm) were used for immunofluorescent staining of Wilms tumor 1 (WT1). Antigen retrieval was done in 10 mM sodium citrate buffer (pH 6.0) for 20 min by heating in a microwave oven. The section was incubated with blocking buffer (Santa Cruz Biotechnology, Dallas, TX) for 1 to 2 h at ambient temperature. The sections were stained with a primary antibody, rabbit anti-mouse Wilms tumor 1 (WT1) (Santa Cruz Biotechnology) overnight at 4°C. The following day, the slides were washed with PBS-Tween 20 (0.1%) and incubated with a secondary

antibody, goat anti-rabbit Alexa-Fluor-594 (Invitrogen, San Diego, CA). Diamino-2-phenylindole (DAPI) (Southern Biotechnology, Birmingham, AL) was used for nuclear counterstaining. 20 glomeruli of each kidney section were counted and the average number of $WT1^+$ podocytes are shown in the graph.[3]

Measurement of Blood fibrinogen

Mouse blood samples were collected in the presence of sodium citrate from the inferior vena cava and platelet-poor plasma was used for measuring fibrinogen concentrations using the Mouse Fibrinogen ELISA Kit (Innovative Research, Novi, MI).

Statistical analyses

Data were analyzed using GraphPad Prism software version 8. For two groups, parametric Student T-test or non-parametric Mann-Whitney U test were used for analyses of statistical significance. One-way ANOVA followed by Tukey's or Dunn’s multiple comparison test for four groups of parametric or non-parametric data. Values were expressed as the mean ± standard error of the mean (SEM).

References

1. Prasad JM, Gorkun OV, Raghu H, et al. Mice expressing a mutant form of fibrinogen that cannot support fibrin formation exhibit compromised antimicrobial host defense. *Blood*. 2015;126(17):2047-2058.
2. Paszty C, Brion CM, Manci E, et al. Transgenic knockout mice with exclusively human sickle hemoglobin and sickle cell disease. *Science*. 1997;278(5339):876-878.
3. Nasimuzzaman M, Arumugam PI, Mullins ES, et al. Elimination of the fibrinogen integrin alphaMbeta2-binding motif improves renal pathology in mice with sickle cell anemia. *Blood Adv*. 2019;3(9):1519-1532.

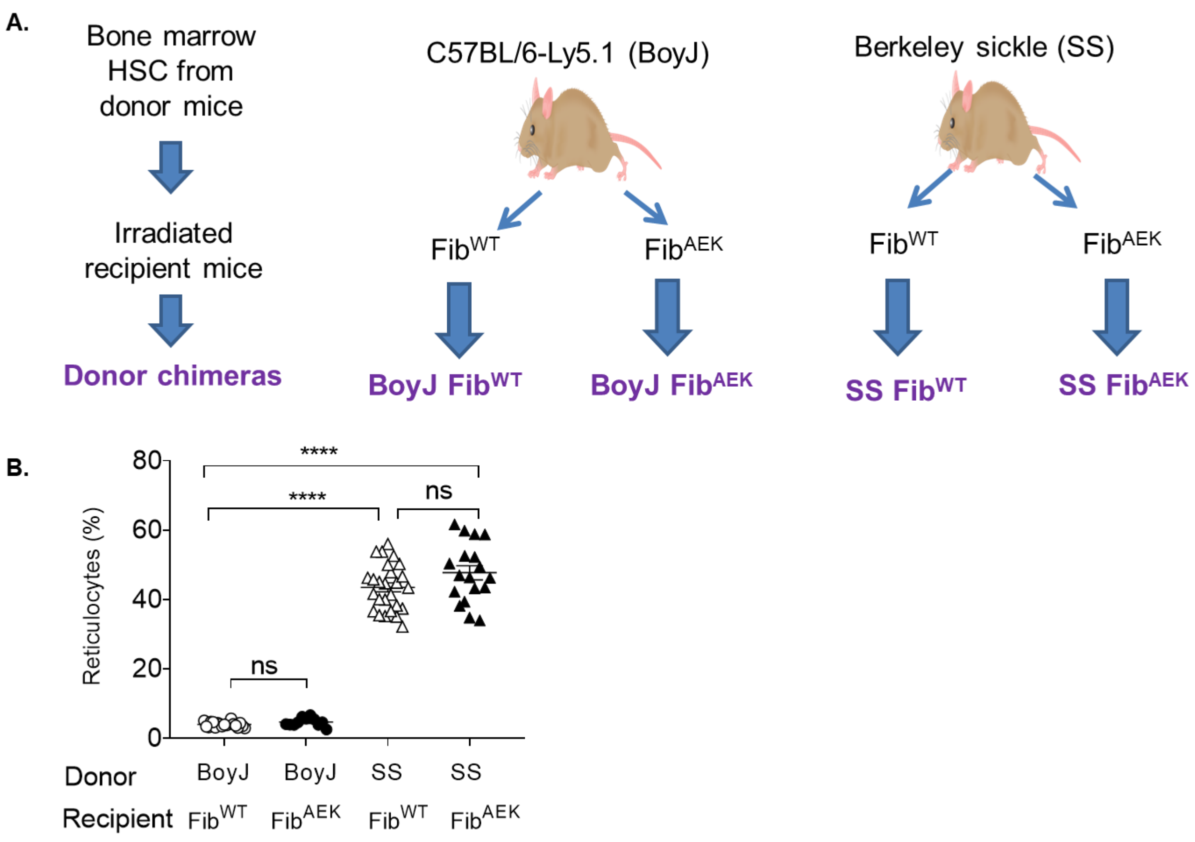

Supplemental Figure S1. The experimental approach to generate sickle RBC chimeras in wild-type fibrinogen (Fib^{WT}) or fibrinogen^{AEK} (Fib^{AEK}) mice. (**A**) Bone marrow cells from 8 to 10 week old Berkeley sickle (SS) mice or C57BL/6-Ly5.1 (BoyJ) mice were used as donors for hematopoietic stem cell transplant into lethally irradiated 8 to 10-week old Fib^{WT} or Fib^{AEK} recipient mice. (**B**) Percentages of reticulocytes in the BoyJ Fib^{WT} (n=19), BoyJ Fib^{AEK} (n=14), SS Fib^{WT} (n=26), and SS Fib^{AEK} (n=20) at 12 months post-BMT. Each symbol represents an individual mouse. The bars in the graph indicate the mean ± SEM. Statistical significance was determined using one-way ANOVA followed by Dunn's multiple comparison test. Statistical significance is indicated as ****P≤0.0001; ns, not significant.

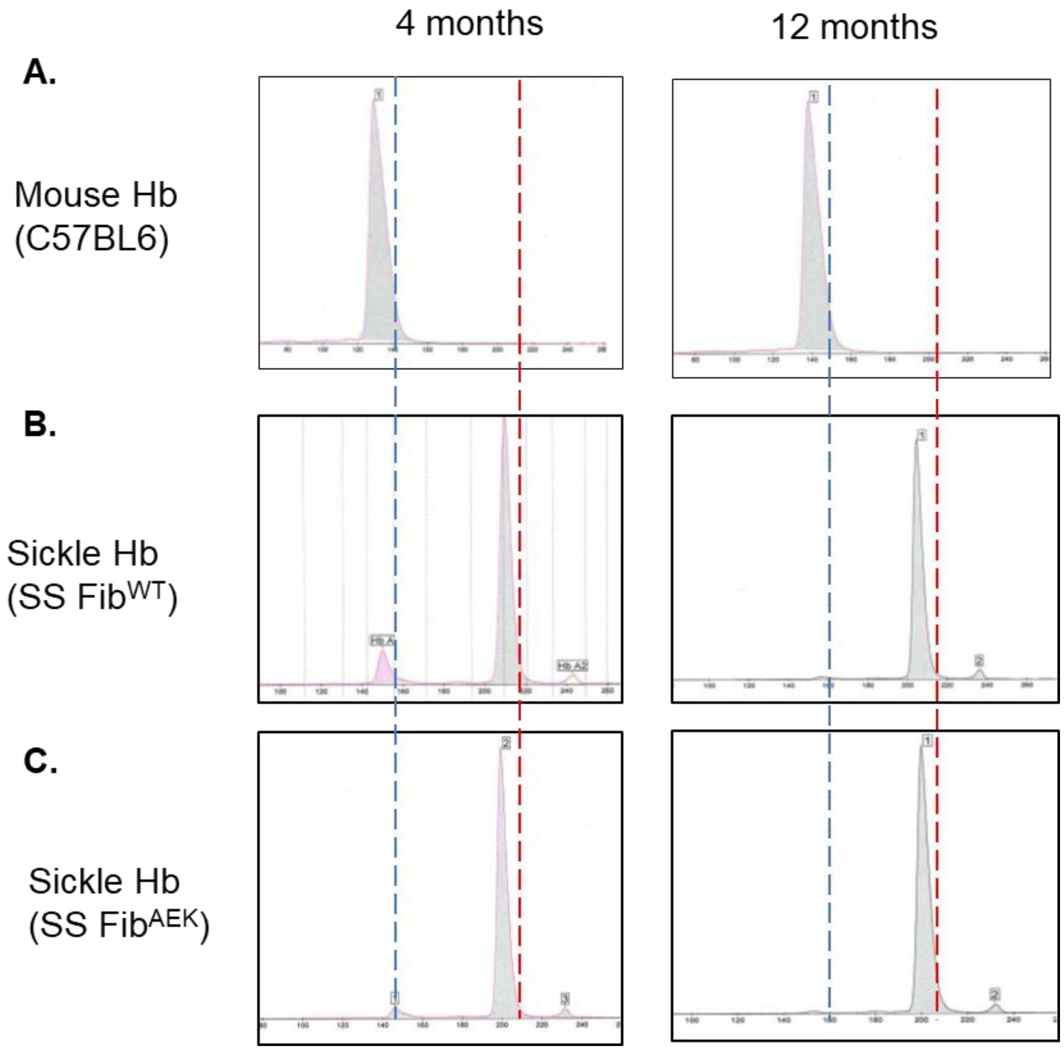

Supplemental Figure S2. Analysis of human sickle RBC hemoglobin (Hb) chimerism in the SS Fib^{WT} and SS Fib^{AEK} mice. Bone marrow cells from 8 to 10 week old Berkeley sickle (SS) mice were used as donors for hematopoietic stem cell transplantation into lethally irradiated 8 to 10-week old Fib^{WT} and Fib^{AEK} recipient mice. (**A**) Capillary Zone Electrophoresis (CZE) profile of RBC lysates from C57BL/6 mouse showing electromigration curve of mouse adult hemoglobin at 4 months of age (left curve) and 12 months of age (right curve). (**B**) CZE profile of RBC lysates from a SS Fib^{WT} mouse showing electromigration curve of human sickle hemoglobin at 4 months post-BMT (left curve) and 12 months post-BMT (right curve), (**C**) CZE profile of RBC lysates from a SS Fib^{AEK} mouse showing electromigration curve of human sickle hemoglobin at 4 months post-BMT (left curve) and 12 months post-BMT (right curve). The positions of mouse hemoglobin are marked with a blue line and the positions of human sickle hemoglobin are marked with a red line.

Supplemental Table S1. Hematologic parameters of the BoyJ Fib^{WT} (n=19), BoyJ Fib^{AEK} (n=14), SS Fib^{WT} (n=26), and SS Fib^{AEK} (n=20) mice at 12 months post-BMT.

Parameter	BoyJ Fib^{WT}	BoyJ Fib^{AEK}	SS Fib^{WT}	SS Fib^{AEK}
Hemoglobin (g/dL)	11.8 ± 0.4	11.1 ± 0.5	8.0 ± 0.2****	7.8 ± 0.2****
RBC (10^6/µL)	8.5 ± 0.1	7.8 ± 0.3	6.2 ± 0.1****	5.6 ± 0.2****
Reticulocytes (%)	4.0 ± 0.2	4.7 ± 0.4	43.5 ± 1.3****	49.2 ± 2.3****
RDW (%)	18.3 ± 0.5	19.0 ± 0.5	32.6 ± 0.5****	33.0 ± 0.8****
WBC (10^3/µL)	9.2 ± 0.5	8.5 ± 0.4	12.0 ± 1.1****	13.1 ± 1.2****
Neutrophil (10^3/µL)	2.0 ± 0.3	1.8 ± 0.2	2.7 ± 0.3	4.0 ± 0.4***
Monocyte (10^3/µL)	0.5 ± 0.1	0.5 ± 0.1	0.9 ± 0.1*	0.9 ± 0.1*
Lymphocyte (10^3/µL)	6.8 ± 0.4	6.1 ± 0.4	8.0 ± 0.9	8.1 ± 1.0
Platelets (10^3/µL)	1320.6±91.3	1285.9 ± 73.8	1038.3 ± 30.4*	1082.2 ± 66.8

RBC, red blood cells; WBC, white blood cells; RDW, red cell distribution width. Data are presented as Mean ± SEM. Statistical significance was determined using one-way ANOVA followed by Tukey's or Dunn's multiple comparison test. Asterisks indicate statistical significance compared with BoyJ Fib^{WT} mice within the same experimental group (*P, ≤0.05, ***P, ≤0.001, and ****P, ≤0.0001). There were no statistically significant differences in parameters between BoyJ Fib^{WT} vs BoyJ Fib^{AEK}, and SS Fib^{WT} vs SS Fib^{AEK} mice.

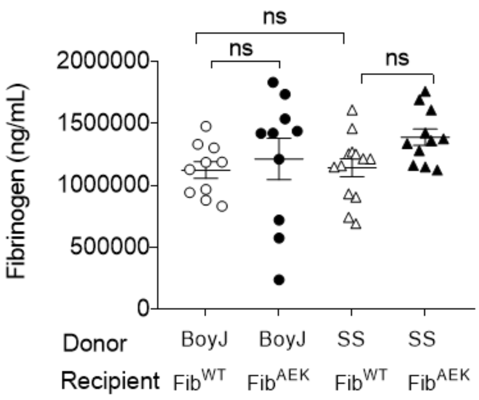

Supplemental Figure S3. Analysis of blood fibrinogen of the experimental mice. Platelet poor plasma samples were used for measuring fibrinogen from the BoyJ Fib^{WT} (n=10), BoyJ Fib^{AEK} (n=10), SS Fib^{WT} (n=13), and SS Fib^{AEK} (n=10) mice at 12 months post-BMT. Fibrinogen concentrations were similar among all groups of mice. Each symbol in the graph represents an individual mouse. The bars in the graph indicate the mean ± SEM. Statistical significance was determined using one-way ANOVA followed by Tukey's multiple comparison test. Statistical significance is indicated as ns: not significant.

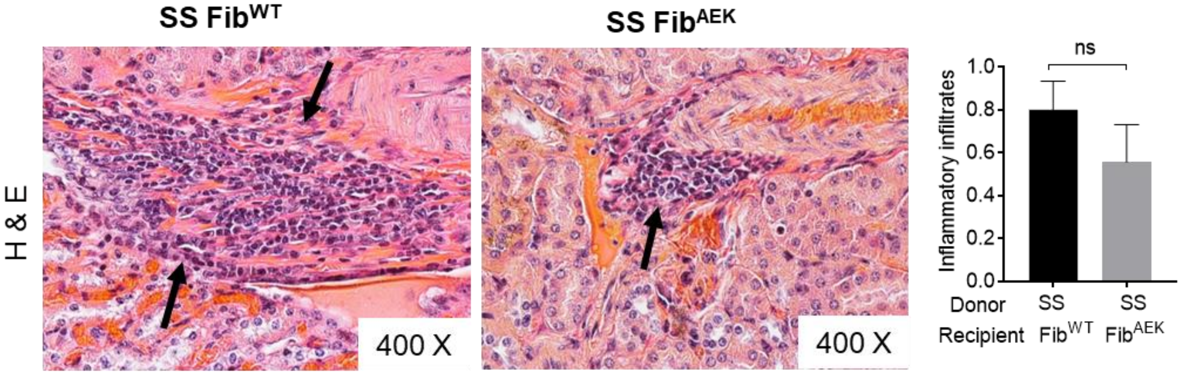

Supplemental Figure S4. Inflammatory infiltrates in SS Fib^{WT} vs SS Fib^{AEK} mice. Representative kidney sections of SS Fib^{WT} mice (left panel), Fib^{AEK} SS mice (middle panel), and semi-quantitative score of histologic features (right panel) showing inflammatory infiltrates at 12 months post-BMT. Arrows indicate inflammatory infiltrates. Each kidney section was entirely examined and scored. Histopathology scores are ranged from 0 to 5, where 0 is the normal kidney morphology; 1 is the pathology in less than 20% the kidney sections; 2, is the pathology in 21% to 40% the kidney sections; 3, is the pathology in 41% to 60% the kidney sections; 4 is the pathology 61% to 80% the kidney sections. The bars in the graph indicate the mean ± SEM. Statistical analyses of the histologic scores were done by Mann-Whitney U test. Statistical significance between SS Fib^{WT} (n=10) and SS Fib^{AEK} (n=9) mice is indicated as ns: not significant.

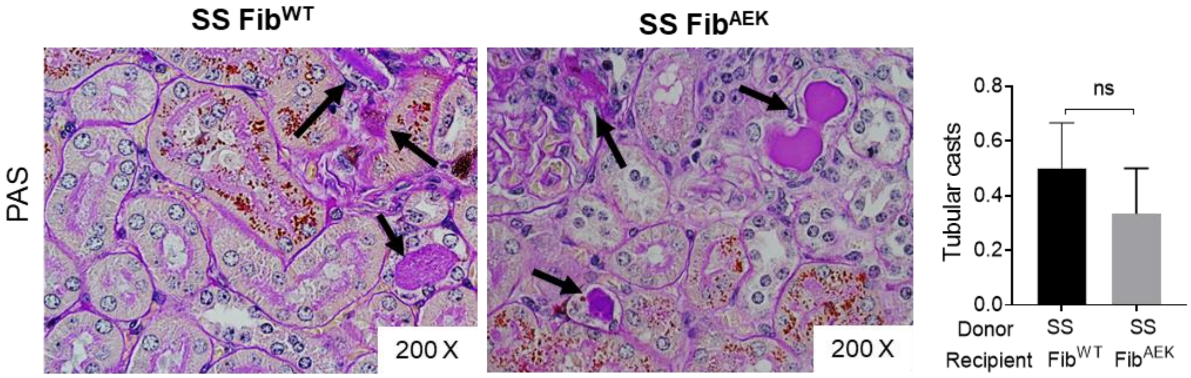

Supplemental Figure S5. Tubular pathology in SS Fib^{WT} vs SS Fib^{AEK} mice. Representative kidney sections of SS Fib^{WT} mice (left panel), Fib^{AEK} SS mice (middle panel), and semi-quantitative score of histologic features (right panel) showing tubular pathology at 12 months post-BMT. Arrows indicate tubular casts.). Histopathology scores are ranged from 0 to 5, where 0 is the normal kidney morphology; 1 is the pathology in less than 20% the kidney sections; 2, is the pathology in 21% to 40% the kidney sections; 3, is the pathology in 41% to 60% the kidney sections; 4 is the pathology 61% to 80% the kidney sections. The bars in the graph indicate the mean ± SEM. Statistical analyses of the histologic scores were done by Mann-Whitney U test. Statistical significance between SS Fib^{WT} (n=10) and SS Fib^{AEK} (n=9) mice is indicated as ns, not significant.

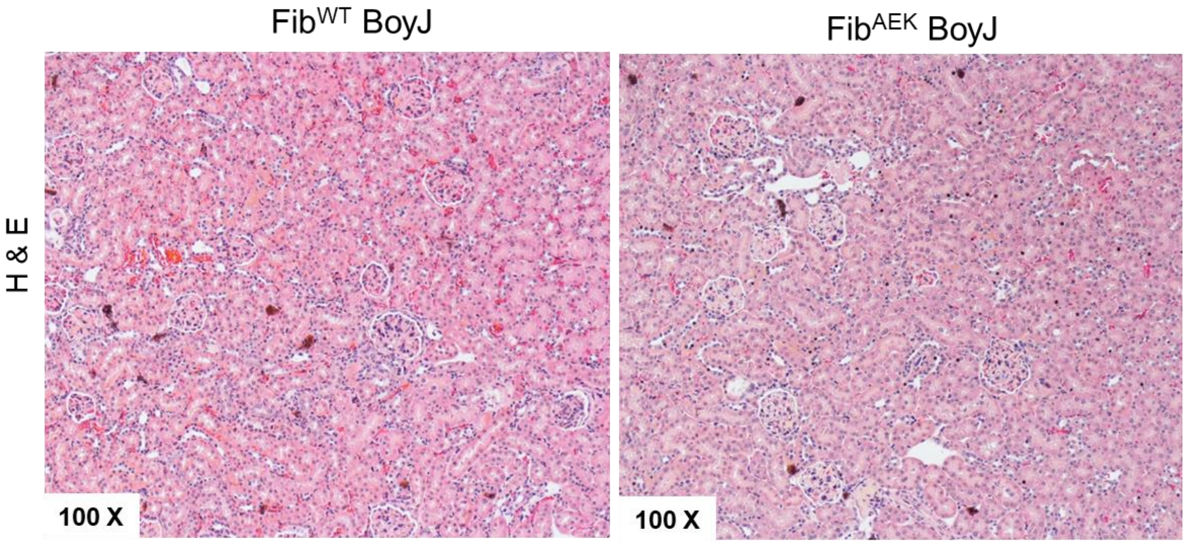

Supplemental Figure S6. Non-SS kidney sections of BoyJ Fib^{WT} and BoyJ Fib^{AEK} mice do not show any pathology. Non-sickle mice were followed for 12 months post-BMT and kidneys were fixed in formalin after euthanizing the mice and stained with H & E reagents.
